# Parents of preterm-born children; sources of stress and worry and experiences with an early intervention programme – a qualitative study

**DOI:** 10.1186/1472-6955-12-28

**Published:** 2013-12-06

**Authors:** Nina M Kynø, Ingrid Helen Ravn, Rolf Lindemann, Nina Aarhus Smeby, Anne Mari Torgersen, Tonje Gundersen

**Affiliations:** 1 Department of Research and Development, Division of Emergencies and Critical Care, Oslo University Hospital, P O Box 4956, Nydalen, NO-0424 Oslo, Norway; 2 Faculty of Social Science, Department of Psychology, University of Oslo, Oslo, Norway; 3 Lovisenberg Diaconal University College, Faculty of Post-graduate Nursing Education, Oslo, Norway; 4 Department of Nursing, Oslo and Akershus University College of Applied Science, Faculty of Health Science, Oslo, Norway; 5 Department of Neonatal Intensive Care, Women and Childrens´ Division, Oslo University Hospital, Oslo, Norway; 6 Faculty of Medicine, University of Oslo, Oslo, Norway; 7 Unit of Research, Innovation and Education, Oslo University Hospital, Oslo, Norway; 8 Department of Nursing and Health Science, Faculty of Medicine, University of Oslo, Oslo, Norway; 9 Norwegian Social Research (NOVA), Oslo, Norway

**Keywords:** Early intervention, Focus group interviews, Neonatal intensive care unit, NICU, Parental stress, Preterm infant, Public health nurses, Thematic analyses

## Abstract

**Background:**

Preterm-born children are at increased risk of adverse developmental outcomes, and their parents may experience increased stress levels. The Mother–Infant Transaction Program (MITP) is an early intervention that aims to enhance the parent–infant relationship and child development. The present study investigated differences in parents’ experience of stress and concerns about caring for their preterm-born child according to whether they participated in the programme. Parental satisfaction with the intervention was also explored.

**Methods:**

As part of a follow-up study at 36 months, a randomized controlled trial of the MITP—14 parents of 11 children from the intervention group, and 17 parents of 14 children from the control group were interviewed by the use of semi-structured focus group interviews. The interviews were analysed thematically.

**Results:**

The intervention parents reported that the knowledge, advice, guidance and emotional support given during the intervention made them feel less stressed and more confident, competent and secure caring for their preterm born child than they would otherwise have been. The control parents described feeling less involved and emotionally supported, and seemed more anxious about their child’s development than the intervention parents. All parents were vigilant and alert to their child’s needs and monitored developmental milestones carefully.

**Conclusion:**

This qualitative exploration of the influences of the MITP revealed a positive impact of the intervention and seems to be an important educational and supportive initiative. Thus, reducing parental stress and enhancing confidence in the parental role.

## Background

Parents of preterm-born infants often experience an increased level of parental stress [1-3] and emotional burden, which can last for years [4,5]. Parents’ concerns are reasonable because children born preterm at a gestational age of less than 37 weeks are at increased risk of subsequent adverse outcomes in various aspects of development [6-8].

Compared with term infants, preterm infants are born with an immature brain and nervous system [9]. Neonatal nurses caring for preterm infants in the neonatal intensive care unit (NICU) observe that infants with low gestational age interact less through prolonged eye contact, make fewer grimaces and respond less to interaction compared with term infants. This behaviour can also continue after discharge. The preterm-born infant’s nervous system overloads easily, and the light, noise and painful procedures in the NICU environment add to the infant’s exhaustion [10,11]. Because most of the preterm infant’s energy is used to maintain essential physical functions such as breathing, circulation, body temperature and digestion, little energy is left for social interaction and transaction between parents and child. Moreover, social interaction may burden their immature nervous system additionally and cause disorganized and poor regulation and stressed behaviour. The preterm infant’s signals and behaviour are often unpredictable and difficult for parents to interpret and respond to appropriately [12]. Thus, characteristics of the preterm infant combined with parental stress can lead to unfavourable transactions between the parent and child.

In 2005, a study group at Oslo University Hospital, Norway, implemented an early intervention programme aimed at training parents how to interact with and care for their preterm infant. A randomized controlled trial was conducted to measure the effects on outcomes in parents and preterm infants during the first 12 months [13]. The present study is a part of the follow-up study of some of the same children and parents at 36 months [14].

Various early interventions to enhance preterm-born children’s development and/ or the parent–infant relationship have been initiated. These interventions differ in their timing, hours of intervention, focus and content [15,16].

Some interventions are implemented during hospitalization; e.g., the Newborn Individualized Developmental Care and Assessment Program [15,17], and kangaroo care, which involves skin-to-skin contact between mother and infant [18,19]. Others are post-discharge interventions; e.g., the Infant Behavioural Assessment and Intervention Program, which focuses on the infant’s self-regulatory competencies and developmental functions [20]. Other pre- and post-hospitalization interventions seek to bridge the transition to home; e.g., the Creating Opportunities for Parent Empowerment [21] and the Mother–Infant Transaction Program (MITP) [22].

The study group at Oslo University Hospital tested the effects of the MITP, which has been reported to have positive effects on maternal experiences of their parenting role, parenting stress and child development [23]. The intervention focuses on both child development and the parent–infant relationship, and builds upon the theory of mutual transaction in the parent–infant relationship. Child development is seen as a result of the transaction between the parents and infant, and experiences in the infants’ environment [24]. The aim of the MITP is to teach the mothers (and fathers) how to be sensitive and responsive to their infant’s physiological, behavioural and social cues; to help the infant remain within an emotional-coping range; to aid the infant’s emotional self-regulation; to reduce both the infant’s and parents’ stress; and to enhance child development and parental adjustment. The MITP is a semi-structured intervention programme comprising 11 one-hour sessions with each mother (and father) and child [12]. The intervention nurse teaches and guides the parents individually and the parents can ask questions and get feedback in how they assess and handle the infant.

The first seven sessions took place at the hospital, with the individual parents in a separate room in the NICU, during the last week before discharge. In the first introductory session the Brazelton Neonatal Behavioural Assessment Scale was conducted, parents’ reactions and feelings related to having a preterm infant were discussed and they were informed about the infant’s uniqueness and developmental potential. The remaining six sessions focused on teaching the parent(s) to maintain the infant’s physiological homoeostasis in an organized and comfortable state, and to notice the infant’s signs of distress and disorganization versus signs of composure and stability. The nurses also demonstrated how the infant’s motor behaviour can be signs of disorganisation and how to help the infant into a regulated pattern. To promote social interaction, the parents learned when to respond to the infant’s cues so as to reduce stress and promote organization. They also learned how to assess different levels of consciousness and to regulate sleep in order to help the infant to regulate him- or herself, and to promote and sustain social interaction. To prepare for discharge, the intervention also focused on how to provide daily care in an effective way. The last four sessions occurred during home visits at 3, 14, 30 and 90 days after discharge, and these focused on adjustment to the domestic environment, mutual enjoyment through play and the concept of child temperament. In the last session, the intervention nurse assessed the infant’s development [12].

Formally trained neonatal nurses led the intervention, and all sessions with the mother (father) and infant. The intervention nurses followed prewritten instructions and wrote a detailed log-book for each session, and a research assistant (first author) supervised the whole process. During the sessions the nurses were instructed to answer all questions from the parents but not to introduce new themes. Regular counselling with the intervention nurses and the leader of the MITP project was also included to maintain fidelity to the programme.

During the two years when the intervention was carried out all participants also received standard care at the NICU, including unlimited visiting hours for parents (except during medical rounds and nursing documentation), the use of kangaroo care and nursing initiatives such as nesting the infant, clustering of procedures, shielding of incubators, toning down light and noise and giving information and guidance to parents.

Different quantitative studies have been initiated to measure the effects of the MITP on parents. Studies have found that the MITP contributed to enhanced mother–infant interactions [25,26], particularly among first-time mothers [13]. Rauh et al. [22] found increased satisfaction among mothers in the intervention group regarding the mothering role, and Ravn et al. reported that they had less post-partum depression 1 month after discharge and breastfed their infants for longer [27]. At 12 months of age, the intervention mothers scored higher than did mothers in the preterm control group on maternal sensitivity/ responsiveness, and dyads between first-time mothers and infants evinced a higher level of synchrony. More positive mood and less negative mood were also observed among their infants [13]. Parents’ experience of stress in relation to parenting has been measured, with positive effects have been found in mothers at six months, and in both mothers and fathers when the children were 12 months [28] and two years [29] of age.

Although these studies used standardized assessments and have provided important knowledge, we have limited insight into the particular experiences that may cause parental stress and worries in relation to raising a child born preterm. We need a better understanding of how the MITP helps parents to adapt to their parental roles.

In contrast to earlier research, this study analysed the parents’ own accounts of their experiences with the MITP. Our study thereby provides insight into some aspects that causes stress and concern for parents, and how the intervention can be helpful.

A group of the parents who participated in the follow-up study at 36 months was interviewed. The analyses are based on a comparison between the experiences of parents of preterm-born children who participated in the MITP and parents of preterm-born children who received standard care only at the NICU, as assessed through focus group interviews.

The aim of the present study was to explore any differences between the intervention and control groups in terms of how the parents describe their experience with stress and worries while raising a preterm-born child. We also aimed to explore the parental user perspectives of the MITP when the children were about 36 months of age.

## Methods

### Focus group interviews

The analyses in this article are based on data from semi-structured focus group interviews with 31 parents of 25 children. The interviews were part of a follow-up study of a randomized controlled trial [14]. The group of parents who participated in the MITP intervention (the intervention group) comprised 10 mothers and four fathers of 11 children. The group of parents who had received only standard care (the control group) comprised 11 mothers and six fathers of 10 singletons and two pairs of twins. Initially, the parents were free to choose if both parents or only one parent would take part in the interviews.

In the semi-structured interviews, the participants (both intervention and control) were asked to discuss: if they thought that parents of preterm-born children would experience more stress and worries in their role compared to parents of children born at term. In addition, the intervention parents were asked to give positive and negative feedback about their experiences with the MITP. Open questions were asked to make it possible for the parents to start the discussion themselves and discuss the themes they found most interesting. This approach was successful because the parents immediately started to reflect on and discuss their own experiences.

An experienced neonatal nurse with special skills in family dynamics moderated the groups. She had not previously met the participants. The first author was present as an observer during the five focus groups – two with the intervention group and three with the control group. Each group comprised 4–7 participants. In addition, one parent from the intervention group was interviewed alone because other parents in this group did not attend the scheduled meeting and it was not possible to form another focus group. The interviews were limited to 90 minutes, and each lasted 80–90 minutes.

The strength of focus group interviews is the group process, the interaction and the participants’ discussion, which can provide information relevant to the purpose of the study. Participants can share stories and comment on each other’s experiences, which can produce more detailed information and a deeper perspective [30]. The focus group interviews allowed us to explore the parents’ experiences related to caring for their preterm-born child and their experiences with the health services, and gave insight into which aspects of the intervention programme that were particularly salient to parents.

### Participants

The infants of the participating parents were single-born infants and twins born in 2005–2006, with a gestation age from 30.0 to less than 36.0 weeks. They were all born at Oslo University Hospital, and were all hospitalized for more than 8 days in the NICU [13]. None of the infants had congenital anomalies, hearing loss or chromosomal disorders. The mothers recruited to the study had to live in Oslo; speak, read and write Norwegian; and have no previous history of alcohol or drug abuse or psychiatric disorder. After the parents had signed an informed consent form, computer-generated random numbers allocated the infants to the intervention or control group. Twins were allocated to the same group.

In the overall follow-up study at 36 months, the parents of 62 infants participated. All participating parents were invited by mail to participate in the focus group interviews. Thirty-one parents of 25 children decided to participate by giving a positive response and signing the informed consent form.

In the present study, the infants’ mean gestational age was 33.5 weeks in the intervention group, and 33.2 weeks in the control group. The mean birth weight was 2141 g in the intervention group and 1936 g in the control group. There were no significant statistical differences between the two groups. The children had no major sequelae after preterm birth such as intraventricular haemorrage, periventricular leucomalacia, broncho-pulmonary dysplasia, cerebral palsy, hearing or vision loss and can be characterized as a rather healthy group of preterm infants.

The mothers participating in the interviews were on average 33 years at time of birth and had an average of 16.6 years of education; all except one were married or a co-habitant. There were no statistical between-group differences in the mothers’ age at birth, years of education or marital status. Five mothers in the intervention group and none in the control group had former experiences with having a preterm-born child.

The mothers participating in the interviews were on avrage three years older compared to those who declined to participate and this was a statistically significant difference (*p* = .005); they had also 1.4 years more education (*p* = .01) compared to those who did not participate in the interviews.

For the demographic data presented here, SPSS version 18 (IBM Corporation, Somers, NY) was used for the statistical analyses.

### Procedure and setting

The interviews took place in January and September 2009 and January 2010. The intervention parents and control parents were interviewed separately.

A semi-structured interview guide was used to ensure that all groups discussed the same topics, although free discussion about issues that parents wanted to talk about was also permitted. The interviews started with an open question and the interviewer was prepared to move the group towards more structured questions if the participants did not initiate discussion about the topics of interest. However, all parents participated in the discussions during the interviews, and the groups spontaneously covered most of the areas of interest in the research question without the interviewers having to use the probes that had been planned as prompts.

When the parents were asked if they thought, as parents of a preterm-born child, that they experienced more stress or worry in their parental role than did parents in general, they were free to interpret the concepts of “stress” and “worry” as relevant to their experiences. The intervention groups were asked to give positive and negative feedback about their experiences with the MITP. To increase the validity, the moderator confirmed that the answers were understood by summing up or asking, “Did I understand you correctly ….?” The interviews were audiotaped and transcribed verbatim.

### Analyses

The interviews were analysed thematically in six phases as recommended by Braun and Clark [31]. Familiarization of the data was performed through repeated reading, listening and use of notes taken during the interviews in order to attribute each voice to the correct parent, to note the intensity of comments and to start generating the initial codes [32]. Thereafter, the first and the last author identified the codes by searching for themes. The themes were reviewed, organized and assigned to overarching themes before the report was produced. Themes about stress and worry were driven by our theoretical knowledge of parenting stress [31]. Because no previous study has investigated parental user satisfaction and the parents’ experiences with the MITP, we used an inductive approach for this theme. The use of inductive approaches is recommended in new realms of research. The interviews from the intervention groups were analysed in one block, and the interviews from the control groups in another block, so that we could compare the results and investigate group differences.

To increase the reliability, the transcriptions were controlled for accuracy against the audiotapes, and the audiotapes were cross-checked against the transcriptions. The first and the last author analysed all of the interviews independently and discussed their analyses and themes until they achieved agreement. Using the same process they selected quotes according to whether they were most significant for the majority of either control parents, intervention parents or both groups. The selected quotes were used to represent parental thoughts and opinions, and discussed until there was agreement between first and last author.

### Ethical considerations

This research was conducted in accordance with the Declaration of Helsinki. The hospital’s Privacy Protection Supervisor and the Regional Committee for Medical Research Ethics approved the study. The hospital’s Clinical Monitor monitored and approved the quality of the data and the signed consent forms.

## Results

### Hospitalization – a stressful setting

After the birth of their preterm infant, the parents wanted to spend as much time as possible with the infant, and thus divided their time between the NICU and the hospital hotel where they lived. Parents in both groups experienced hospitalization as stressful, as typically expressed by one of the mothers:

“The most stressful time was here at the hospital. To feed my baby and myself — back and forth. Never able to relax and enjoy my baby. The chase between the hotel and the NICU was the worst, and most stress related.”

Separation from the baby at birth and external factors such as busy health professionals or the crowded hospital were conditions that contributed to the feelings of stress among all parents.

Being an inexperienced first-time parent was perceived by both groups of parents as particularly stressful. However, those in the intervention group who had childrearing experience emphasized how the MITP had helped them to reduce their stress, as expressed by one of the mothers regarding her contrasting experiences:

“… We saw a very big difference. Both of our preterm-born children had the same problems. They were vulnerable and needed a lot of close contact. With our first child we didn’t know how to respond. When I had my second preterm baby and got advice from the nurse, I almost cried because I realized how many mistakes I had made with the first. … The advice we got was really helpful … and the reason that it was so much better with the second was not, I think, because we had experience, but simply because we got so much good advice during the intervention that worked.”

All the parents who had previous experience of having a premature child said that they wished that they had this knowledge when they had their first child. However, some mothers reported that some of the advice they received in the NICU when they had their first child (without the intervention) was the same as the advice they received through the MITP.

Parents in the control group said that receiving standard care such as supervision, guidance and the use of kangaroo care decreased their feeling of tension. However, one stress-inducing condition was that they could not spend enough time with their baby. Another factor that led to a feeling of stress was not being recognized or “seen” by the hospital staff as adults with needs separate from those of their babies, as exemplified by one of the control mothers:

“… but I think that it would have helped me if there had been more focus on the parents, too, not only the baby. They took good care of my infant, but I think that a few reassuring sentences each day would have helped me, or if someone had made eye contact with me and had not spoken to me using a child’s language (they usually talked to me and my baby at the same time, as a unit) but had spoken directly to me, as an adult and mother, stressed and worried, with milk burst and God-knows-what; I needed the nurses in neonatal intensive care or … someone who could say, ‘It will be okay.’”

Close follow-up of the parents is an important aspect of the MITP. The findings indicate that being followed-up can be important for parents ability to develop a sense of competence and sercurity related to thir parenting role, as stated by one intervention mother:

“Of course, we were taught some handy tricks and things to do, but to be seen and to have one-to-one guidance and be followed up was very reassuring and nice. The most positive thing was just that feeling that you were followed up so much; it was just unbelievable, a real luxury. The nurse was an amazing person, and we became confident in the parental role faster.”

### Coming home

Coming home was experienced as both a relief and a stressful time for all parents. The parents’ feelings of tension reflected their uncertainty about how to take good care of the infant and how to interpret the infant’s behaviour. The intervention group received home visits and continued to meet the experienced neonatal nurses after discharge, which seemed to ease the potential pressure. By contrast, the control group did not have the same opportunity to consult professionals because their relationship with the NICU staff ended when they were discharged from hospital. Thus, they did not have the opportunity to be guided by knowledgeable intervention nurses, nor did they have the same knowledge as the intervention group had acquired during hospitalization. For instance, one group of control parents discussed how the infants were inaccessible and how this made it more difficult to make contact and interpret the infant’s signals. In accordance with the theoretical rationale of the MITP, one of the control mothers gave an account of the stress she was felt related to the inaccessibility of her preterm infant:

“… I have a second baby now — he came at term and the interaction between us is very different. … With our preterm, it took time to make contact or achieve an interaction. He was this baby who was in his own world … who should have been in the womb for 2 more months. I noticed very well when he slept, he dozed a lot, he cried too, but sometimes he was also half asleep, he smiled only after a long time and it was very difficult to interpret his signals: Is he hungry? Is he tired? Is he hurt? Whereas with a child at term, I think they provide clearer signals to the mother about what’s wrong, what they want; so I personally experienced more stress in the mother’s role for my preterm baby…”

The equivalent discussion in the intervention groups was about how preterm infants differed from term infants, but also about the effectiveness of the advice they received. In contrast to the control parents, the intervention parents talked about how they had learned about the preterm infants’ different levels of consciousness and accessibility for interaction, and their capacity to maintain interaction. Parents explained how they had tried out what they learned and had experienced that it worked; and as a consequence, their feeling of competence had increased.

Like all Norwegian parents, the parents in this study were followed up by the public health nurse after going home. The follow-up usually comprises one home visit and regular consultation with the public health nurse at the well-baby clinics. In both intervention and control groups, parents felt that the public health nurse rarely had adequate knowledge about prematurity and therefore was unable to support and calm parents. The parents noted that the public health nurses focused primarily on weight gain and growth, and lacked a focus on other developmental issues distinct to preterm infants or on the parents’ emotional needs in this situation. The public health programme is designed for term infants and is delivered by public health nurses and the well-baby clinics did not adequately meet the individual needs of the parents in this study. Because of these experiences some parents said that the public health service was a waste of time, as one mother said:

“… I did not bother to use my time to visit the well-baby clinic; it is always the same, ‘But everything is normal’… everything is probably normal too, but when I ask a question and am feeling a ‘little’ worried, it would have been nice if they had a little more to say than ‘Everything is normal.’”

Failure of parents of preterm infants to use the public health service because they find it useless is important information for the public health nurse whose assignment is to monitor the general health of infants and children^a^. This failure affected the control parents in particular because they had no other assigned professionals to whom they could turn. The intervention parents, by contrast, had home visits from the intervention nurse, and her competence and interest gave them a feeling of security. Knowing that they had someone to ask at some point, if not “right now”, was perceived as stress reducing, as one intervention mother said:

“… and I thought, ‘Now the intervention nurse will come soon,’ and this gave me a feeling of security, that we could ask some additional questions, get some tips and advice.”

In addition to calming parents by helping them with their childrearing tasks, the intervention nurse helped parents abate feelings of anxiety. Except for the opening question during the first session, the MITP does not include a focus on parental debriefing. However, after coming home parents may feel a strong need to talk to a professional about their experiences – to debrief. In contrast to the control parents, the parents in the intervention group talked about their opportunity to talk about their expereiences at the hospital, their anxiety regarding the child, and other issues of concern.

In general, the intervention content, guidance and counselling were experienced as vital, providing information and practical tips that the parents appreciated and benefited from. One father summarized his experience with the MITP as follows:

“I think probably we have learned something that is beyond knowledge just about the baby, although I felt that most [of the programme] was directed towards it.”

Obtaining knowledge made it easier for the intervention parents to accept new challenges, but also gave them a tool and security for improvising. Parents who later had another preterm-born child, told how they successfully applied what they had learned from the intervention to a new child, as one intervention mother said:

“When we had another premature child I thought, God, I’ve been so lucky, you know, it was really like, … I could just brush up everything again and use all I had already learned!”

### Parents’ Confidence and concern in everyday life

Finding relevant information about moderate and late preterm-born children was percived as difficult by all the parents in this study. Most of the available information they had found focused on preterm infants of lower gestational ages which they found less useful. Thus, the parents in both groups said it would have been helpful to meet other parents of children born at about the same gestational age as their child. This became evident during the interviews, as the parents stated that they appreciated the opportunity the interviews gave to discuss and share experiences, stories and thoughts with other parents.

One topic discussed in both groups was how they compared their child’s size and development with that of term-born children and how preterm-born child has a longer way to go. They did not take normal development for granted and continuously observed their child as expressed by one mother:

“No, I don’t think you can let go of the [focus on] prematurity completely. When you have a premature child, you don’t take the development for granted like you do with term-born children. With preterms, you are always a little alert in relation to development, like in relation to kindergarten, you are maybe more concerned about a premature child … because you have a notion that he or she has a longer way to go than a child born at term. The term born children are in a way automatically there [at developmental milestones], and I think this mindset will stick with a parent at least until their child starts school. I think maybe the concern will always be there to some extent.”

Because they did not know what to expect about the development of a preterm-born child of their child’s gestational age, each parent wondered about *“When has my child caught up?”* Parents who focused solely on when their child would reach an average developmental level seemed to experience more stress than did those who were aware of different possible development trajectories. However, such feelings were more pronounced among the parents in the control group, than among the parents in the intervention group. One of the control mothers said:

“When I gave birth, health professionals said that a preterm infant catches up with term peers during the first year, so I didn’t need to worry. As we were discharged from the hospital, health professionals said that within a few years his development would level out to the normal range. At some visit to the well-baby clinic, they said that before school starts, the child will have caught up developmentally. And now they say that it is during school age that you can see if there are any developmental disadvantages. I feel it prevailing everywhere, that health professionals portion it out [extending the time frame], so I have in a way probably more worries about just that than about my child.”

The parents from the intervention group seemed more conscious of the importance of not comparing the preterm infant’s development with that of term infants because the preterm’s development may progress differently to that of term-born children.

The MITP’s focus on the child’s developmental potential from the very beginning gives parents an opportunity to discuss prematurity, the infant’s developmental potential, and prematurely born childrens progress extensively with the intervention nurse. Thus, this may explain the intervention parents’ acceptance of differences in developmental trajectories between preterm and term-born children.

Both the intervention and the control parents were aware of the vulnerability of preterm infants to developmental problems and stated that parents of preterm-born children may be more worried than are parents of term-born children as one mother said:

“All parents are concerned about their children, but when the child is born prematurely, this provides an additional concern …”

### Concerned and worried toward alert and vigilant

The interviews indicate that parents stress and worries reduces as the children grow older. The parents said they continually monitored their child’s development, focusing on their developmental milestones while having the child’s prematurity in mind. However, when describing their feelings when the child was a baby, the parents used words like concern and worry. As the children grew older these termes did not seem to capture their experience accurately, and words like “alert or vigilant” seems more suitable as exemplified by this fathers statement:

“Worried may not be the right word; you are perhaps more alert. They might need more follow-up … But, after a while, when I saw that my child had completely normal curves [on the growth chart], I stopped worrying. Nevertheless, I’m certainly more alert — more on my toes, I think … But it’s wrong to say or to use the word worried after the 2–3 first months.”

A mother:

“I kind of agree with you, I think that it is not so much concern, at least not yet, but it’s just that you’re watching certain things and perhaps especially around learning or concentration, and thus, one becomes a bit more sensitive in trying to track such things that you in a way know or have heard or have an idea may be affected by prematurity; you are a little more vigilant about these things.”

### Intervention parent´s recommendations for the MITP

Parents who participated in the MITP said that the intervention should be extended to other health professionals such as the public health nurse. Although the parents stated that parents with preterm-born infants of lower gestational age would benefit and need the intervention even more than they did, they thought that the intervention should be given to all parents of preterm infants.

Most parents who had participated in the MITP felt that the numbers of hours of the intervention was sufficient. However, some parents found it difficult to absorb the information during the hospital stay and said that they would have appreciated receiving additional written information about the intervention and the practical advice given.

## Discussion

Asking all parents about “stress” and “worry” allowed them to reflect freely and to discuss their experiences of having a preterm-born child. The intervention parents were also asked about their experiences of the intervention.

Some control parents discussed the inaccessible behaviour of the preterm infant and their lack of knowledge about how to understand and respond to the child. By contrast, the intervention parents found that the advice they received was useful in the daily care of their child. They noted how this boosted their confidence and feeling of security, in that they had a repertoire of alternatives for actions. They discussed how the tips and knowledge given through the intervention seemed to work when handling the infant. Additionally, because the intervention continued over a period of 3 months, the parents had the opportunity to try out the advice and obtain feedback on how they handled their infant. This shows that the intervention supported the parents in their efforts to interpret their infant’s signals and thereby helped the infant remain in an accessible state and emotional coping range this is one of the main issues addressed in the intervention. The increased parental competence creates positive interaction loops and strengthens the transactions between the infant and parent. This parents´ description confirms the theoretical underpinnings and the main purpose of the MITP, which is to prevent a downward spiral of unfavourable transactions influencing the parent–child interaction pattern before an established pattern arises [12].

Experiences of previously having a former preterm-born child may help parents abate feelings of stress and worry. However, as shown in this study, the parents who had participated in the intervention said that the guidance they received allowed them to feel secure and confident in their parental role faster even when they had previous experience with a preterm child. Being a first-time parent may increase the feeling of stress and may thereby increase the impact of the intervention. This may also explain why first-time mothers in particular showed better mother-infant interaction when the effects of the MITP were assessed at 12 months [13] and why experienced parents wished they had had the MITP when they had their first preterm-born child.

Access to high-quality standard care, for example kangaroo care and guided participation in caring for the infant, may have reduced the experience of stress for all parents and may have lessened the difference between the intervention and control groups. For instance the control group mentioned that kangaroo care reduced stress. Such care has been found to increase a mother’s feeling of control over stress and worry, making her more stress-resilient [33]. However, Tessier et al. suggested that kangaroo care can have an isolating effect and that adding a social support component may be beneficial [33]. Some parents from the control group mentioned that they felt ignored, which may indicate such an isolating effect. “To be seen” implies being treated as an unique person and having someone who listens, comforts, supports, show respect and provides assurance [34], which is supported by the findings of this study. The MITP, however, worked as a social and emotional support because the intervention parents felt that they had been seen and followed up. This kind of support seemed to make the parents less vulnerable to stress in the stressful setting of the hospital. Hence, the MITP worked, as a buffer against what parents may otherwise perceive as “not being seen” by busy health professionals focusing solely on the infant’s needs.

First-time mothers of term infants have rated the public health nurse as an important source of informal support and appraisal [35]. The parents in this study did not find that the public health nurses were able to meet their needs for emotional support and knowledge about their child’s prematurity. The parents needed health care providers whom they could ask questions and who could confirm or correct the way they nursed the infant. The intervention parents knew they had someone to ask — the intervention nurse. However, this also indicates that health care providers should meet the parents’ need for more access to differentiated information appropriate to their child’s specific gestational age as the child grows up. The emotional support that the parents described experiencing at hospital and later at home may explain the reduction of post-partum depression 1 month after discharge observed by Ravn et al. [27].

Emotional support through the intervention was appreciated and seen as an important positive component of the MITP. The parents’ quotations reveal that they used the intervention nurse for emotional support. The first intervention session in the MITP focuses on getting acquainted with the parents and infants and to discuss the parents’ reaction to their infant’s hospitalization [12]. No session was dedicated to emotional support per se, although parents could and did ask the intervention nurse questions and discussed themes of interest with them. Intervention programmes with the objective of preventing psychological trauma after preterm birth can reduce symptoms of trauma [36]. One study of the MITP included modifications such as one additional hour for parents to debrief about their experiences of and feelings about birth and hospitalization [28]. The findings in the present study indicate that modifying the MITP to include this hour may increase the efficacy of the intervention and, importantly, that such a modification may help reduce parental stress even further.

Although the early intervention was experienced as helpful for parents during hospitalization, other aspects of their stay continued to cause stress. A well-known stressor for mothers delivering preterm is the separation from the baby at birth [34,37], and the MITP was not helpful in this respect. One stress-reducing solution may be the rooming-in of parents because this would help them remain close to the infant and avoid the “chasing” between hotel or home and the NICU. During hospitalization in the NICU single-family rooms can provide restfulness and privacy [38]. Therefore, additional interventions such as rooming-in of the parent and infant in the NICU may be necessary to reduce parental stress.

The findings in this study show that parents want to interact with other parents of children of about the same gestational age as their child. Learning that other parents are preoccupied with the same issues and concerns can increase the parent’s feelings of confidence. Studies show that participating in parental support groups facilitated by experienced parents or NICU staff has positive effects on various aspects of emotional and informative needs [39]. As found in this study, parents appreciate the opportunity to share experiences, stories and thoughts during the interviews.

The intervention gave the parents the opportunity to discuss prematurity and child development extensively with the intervention nurse, who also focused on the infant’s developmental potential. The change in parents from being concerned and worried in the beginning to be alert and to monitor their child's development may be sufficient to detect potential challenges or special needs the child may have.

The previous study of this cohort, which used quantitative measures of the “Parenting Stress Index” [40], did not find differences in parenting stress between 6 and 12 months [2,27]. However, the findings in the present study indicate that the intervention can influence parents experience of stress, by providing knowledge and guidance, and emotional support. These are all factors that the parents underlined as important for their feeling of confidence and security raising a prematurely born child. The different findings may relate to the different methods used. When parents have the opportunity to express themselves in their own words, one may get different results than when using a questionnaire that is targeted to a particular outcome.

Although the number of sessions was considered sufficient by the parents in this study, they requested written materials covering the intervention content. This desire for information may reflect the tension that parents experienced during the hospital stay because stress can make it more difficult to learn and remember provided information. One study modified the MITP to provide written materials and found positive effects on various aspects of infant behaviour and development, and maternal stress level [26]. It is therefore plausible that providing parents with written material will increase the efficacy of the programme.

To improve the impact of the MITP, we suggest that the content of the intervention should be retained but that an additional session could be added before the actual intervention starts, for the parents to debrief their experiences of and feelings about the birth and hospitalization. Written materials covering the intervention content should be given to the parents. To bridge the transition between hospital and home, the nurse from hospital should continue the intervention sessions at the parents’ home and also focus on emotional support to the parents. In addition, the intervention content should be disseminated to public health nurses to increase their ability to guide parents of preterm-born infants.

Parents who participated in the interviews felt comfortable with the focus groups. One limitation in using focus group interviews is that parents feel uncomfortable interacting with other parents may not have choosen to participate in the last part of the study. It is possible that the use of one-to-one interviews would have made it easier to schedule interview times to fit parents schedules, which would have made it possible for more parents to attend. The validity of the study may also be threatened if the control parents had educated themselves to incresed knowledge about the content of the intervention. Also the differences in age and years of education between the mothers who participated in the interviews and those who declined may bias the results, because of this attrition we do not know the experiences of the younger mothers.

## Conclusions

This study demonstrates that parents who participated in the Mother–Infant Transaction Program (MITP) experienced a feeling of confidence, competence and security. Such feelings made parents feel secure in their parental roles faster and made new challenges easier to accept. We argue that the parents experiences are related to the knowledge, advice, guidance and emotional support provided to them during the intervention sessions.

Parents who did not receive the early intervention pointed out how they felt less seen and emotionally supported by the nursing staff. They also found that they did not get to spend enough time with their infant in hospital and were to some degree more anxious about the child’s development.

Although the intervention group felt more seen and emotionally supported, the MITP did not eliminate parents’ feeling of stress during hospitalization, and all parents experienced the hospitalization as stressful.

All parents agreed with the statement that parents of preterm-born children experience more stress than do parents of term-born children, but they did not assess themselves as more concerned or worried than other parents, rather more vigilant or alert.

## Endnotes

^a^There is now an ongoing project by Oslo University Hospital to educate public health nurses so that every well baby clinic has one public health nurse with special skills in handling prematurity who is dedicated to parents of preterm-born infants.

## Competing interests

The authors decleare that they have no competing interests.

## Authors’ contributions

NMK made substantive contribution to conception and design, acquisition of data, the analyses and interpretion of the data, drafted the manuscript and approved the final version. IHR made substantive contribution to conception and design, interpretation of the data, revised the manuscript crititcally for important intellectual content and approved the final version. RL made substantive contribution to conception and design of the study and interpretation of the data. NAaS made substantive contribution to conception and design, interpretation of the data and revised the manuscript crititcally for important intellectual content and approved the final version. AMT made substantive contribution to conception and design, interpretation of the data and revised the manuscript crititcally for important intellectual content and approved the final version. TG made substantive contribution to analyses and interpretion of the data and revised the manuscript crititcally for important intellectual content and approved the final version. All authors read and approved the final manuscript.

## Pre-publication history

The pre-publication history for this paper can be accessed here:

http://www.biomedcentral.com/1472-6955/12/28/prepub
